# Computational Amendment of Parenteral In Situ Forming Particulates’ Characteristics: Design of Experiment and PBPK Physiological Modeling

**DOI:** 10.3390/pharmaceutics15102513

**Published:** 2023-10-23

**Authors:** Nada M. El Hoffy, Ahmed S. Yacoub, Amira M. Ghoneim, Magdy Ibrahim, Hussein O. Ammar, Nermin Eissa

**Affiliations:** 1 Department of Pharmaceutics and Pharmaceutical Technology, Faculty of Pharmacy, Future University in Egypt, New Cairo 11835, Egypt; ahmed.yacoub@fue.edu.eg (A.S.Y.); amiraghoneim@gmail.com (A.M.G.); drhusseinammar@hotmail.com (H.O.A.); 2 Bone Muscle Research Center, The University of Texas at Arlington, Arlington, TX 76013, USA; 3 Department of Pharmaceutics and Industrial Pharmacy, Faculty of Pharmacy, Cairo University, Giza 11562, Egypt; magdymohamed1@hotmail.com; 4 Department of Biomedical Sciences, College of Health Sciences, Abu Dhabi University, Abu Dhabi P.O. Box 59911, United Arab Emirates

**Keywords:** in situ forming nanoparticles, parenteral, targeted drug delivery, design of experiment, PDLG, cholesterol, PBPK, lipid, polymer

## Abstract

Lipid and/or polymer-based drug conjugates can potentially minimize side effects by increasing drug accumulation at target sites and thus augment patient compliance. Formulation factors can present a potent influence on the characteristics of the obtained systems. The selection of an appropriate solvent with satisfactory rheological properties, miscibility, and biocompatibility is essential to optimize drug release. This work presents a computational study of the effect of the basic formulation factors on the characteristics of the obtained in situ-forming particulates (IFPs) encapsulating a model drug using a 2^1^.3^1^ full factorial experimental design. The emulsion method was employed for the preparation of lipid and/or polymer-based IFPs. The IFP release profiles and parameters were computed. Additionally, a desirability study was carried out to choose the optimum formulation for further morphological examination, rheological study, and PBPK physiological modeling. Results revealed that the type of particulate forming agent (lipid/polymer) and the incorporation of structure additives like Brij 52 and Eudragit RL can effectively augment the release profile as well as the burst of the drug. The optimized formulation exhibited a pseudoplastic rheological behavior and yielded uniformly spherical-shaped dense particulates with a PS of 573.92 ± 23.5 nm upon injection. Physiological modeling simulation revealed the pioneer pharmacokinetic properties of the optimized formulation compared to the observed data. These results assure the importance of controlling the formulation factors during drug development, the potentiality of the optimized IFPs for the intramuscular delivery of piroxicam, and the reliability of PBPK physiological modeling in predicting the biological performance of new formulations with effective cost management.

## 1. Introduction

Numerous drug candidates who suffer from poor oral bioavailability or minimal half-life now have a higher therapeutic potential, thanks to the development of innovative drug discovery tools, including genetic engineering, combinatorial chemistry, and high-throughput screening [1]. Additionally, these improvements in drug discovery have focused a lot of emphasis on the invention of creative methods to deliver them effectively and efficiently. Long-acting injectable systems are one innovator of such strategies [2]. These systems can sustain therapeutic drug levels for extended periods, offering benefits such as improved bioavailability, consistent plasma concentration, and targeted drug delivery. Furthermore, they are adaptable for various routes of administration, including subcutaneous, intramuscular, and intra-articular, with drug release rates controlled by the formulation’s characteristics [3,4,5]. Both the vehicle and drug characteristics, as well as how the drug interacts with both the tissue and the vehicle, control the drug absorption kinetics and, thus, its duration of action [6].

In systems in which there is positive interaction between the drug and the carrier lipid and/or polymer-yielding implants and/or microparticles, a variety of approaches have been investigated to sustain the drug release. Numerous formulation issues have been observed, including increased process temperature, poor content homogeneity, and the continued requirement for invasive administration in the case of implants in addition to the multi-step manufacturing process and the formulation parameters, which should be closely monitored due to their influence on the scale-up process as well as manufacturing expenses [7].

Initially, approaches that lead to the prolongation of therapeutic activity were examined as viscous oil-based preparations, which could decrease the rate of drug diffusion. Recently, the development of injectable lipid and/or polymer-based particles with biodegradable as well as biocompatible characteristics, exhibiting optimal sizes ranging from 250 nm to 125 nm, has received attention. These particles are used to avoid the discomfort associated with surgical procedures for inserting bulky implants [8]. The latest research focuses on the preparation of a liquid lipid and/or polymer-based formulae containing the drug that solidifies and forms an implant at the site of injection upon contact with body fluids, which represents an effective substitute for conventional solid implants [9]. These formulations are prepared by dissolving polymers as poly(lactide) (PLA) and poly(lactide-co-glycolide) (PLGA) and lipids as cholesterol, phosphatidylcholine, and lecithin in solvents that are miscible with water, such as N-methyl-2-pyrrolidone (NMP) and dimethyl sulfoxide (DMSO). When the lipid and/or polymer solution containing the drug is injected, the lipid and/or polymer converts into an implant by solidification at the injection site [10]. When compared to conventional dosage forms, these cutting-edge formulation technologies have been found to decrease drug clearance and prolong its residence duration. The substantially high initial burst as well as the unfavorable viscosity of these preparations that renders injection inconvenient, are a key disadvantage of such systems. Innovative in situ micro-particles (ISM) were approached after extensive research presenting valid solutions to these issues [11].

ISM dosage forms involve emulsifying the drug-containing internal polymer solution with an external continuous phase of oily or aqueous nature. ISMs are deposited as soon as the emulsified solution comes into contact with the physiological fluids due to the diffusion of the internal phase solvent. These ISM systems greatly minimize the initial burst release as well as the viscosity of the injectable solution, leading to nearly painless injectability and reduced discomfort compared to the outdated polymer solutions. Additionally, ISMs are multi-particulate, which improves the stability as well as the consistency of the release profile of the drug while reducing implant morphological variations [12]. Recently, the application of these multi-particulate systems utilizing lipids as well as non-ionic surfactants is gaining much attention.

Non-steroidal anti-inflammatory drugs (NSAIDs) are the main building block in the treatment of well-known chronic inflammatory disorders via prohibiting cyclo-oxygenase (COX) enzymes. Overdose toxicity is the predominant side effect associated with their clinical usage [13]. While these moieties are mostly administered orally, they cause many systemic drawbacks, which arouses the need for the development of alternative localized formulations [14].

The design of experiments (DOE) is a structured and systematic approach that plays a pivotal role in drug development. In the complex and highly regulated field of pharmaceuticals, DOE is applied to efficiently and comprehensively explore the multifaceted factors affecting drug development processes. It enables scientists and researchers to optimize drug formulations, dosing regimens, and manufacturing processes while minimizing the need for extensive and costly experimentation. By varying and controlling multiple variables simultaneously, DOE can uncover critical interactions and dependencies that impact the safety, efficacy, and quality of drugs. This approach not only accelerates drug development but also enhances the quality of pharmaceutical products, reduces production costs, and ultimately leads to safer and more effective medications for patients. Whether it is the formulation of new drugs, the assessment of potential drug–drug interactions, or the optimization of manufacturing processes, DOE is an indispensable tool in the journey to bring innovative and safe pharmaceuticals to market [15,16].

Physiologically based pharmacokinetic (PBPK) modeling is a vital tool in the realm of drug delivery development. It allows researchers and pharmaceutical scientists to simulate and predict how drugs are absorbed, distributed, metabolized, and eliminated within the human body. Recently, the physiologically based pharmacokinetic (PBPK) modeling approach has grabbed attention in terms of the computational prediction of the different pharmacokinetic parameters of the drug following its administration [17]. PBPK modeling can predict drug concentration–time profiles as well as exposure in blood and specific organs, which are crucial for predicting efficacy/toxicity and risk assessment based on the physicochemical properties of the drug as well as the in vitro and/or ex vivo characterization results [18,19]. In drug delivery, PBPK models are instrumental in designing and optimizing delivery systems to enhance drug effectiveness and minimize side effects. By accounting for factors such as physiological variations, drug properties, and delivery methods, PBPK modeling aids in tailoring drug formulations and dosing regimens for specific patient populations [20]. It guides the development of novel drug delivery systems like nanoparticles, liposomes, and implants, ensuring they reach target tissues or organs effectively. Furthermore, PBPK modeling is indispensable for predicting the pharmacokinetics of sustained-release formulations, intravenous infusions, and transdermal patches, ultimately contributing to safer and more efficient drug delivery strategies, personalized medicine, and the successful translation of drug delivery innovations from the laboratory to clinical practice [21]. The obtained model through PBPK physiological modeling ultimate technological advancement should be validated through its correlation to the published clinical pharmacokinetic data prior to implementation [22]. Pharmaceutical research is currently visualizing PBPK modeling as a useful tool for decision-making at different stages of drug development [23].

The goal of the present work was the computational study of the influence of the various preparation factors on the characteristics of the obtained in situ-forming particulate formulations (IFPs) using the design of experiments (DOE) for the parenteral formulation of a model NSAID (piroxicam). Design Expert^®^ 11 (Stat-Ease, Minneapolis, MN, USA) was used for the creation and analysis of the 2^1^. 3^1^ full factorial experimental design. Furthermore, the release profile of the obtained formulae, as well as their kinetic models, were investigated. The optimum formulation was further investigated morphologically, and the obtained data were correlated to compute their expected biological performance using PK-Sim physiological modeling.

## 2. Materials and Methods

### 2.1. Materials

Piroxicam (PX) was kindly gifted from Medical Union Pharmaceutical (MUP) Co., Egypt. Polyethylene sorbitan monooleate (Tween^®^ 80), Freund’s complete adjuvant (CFA), Sorbitan monooleate (Span^®^ 80), Cholesterol Brij 52^®^, and cellulose membrane dialysis bags were obtained from Sigma-Aldrich Chemie GmbH, Steinheim, Germany. Captex^®^ GTO was kindly donated by Abitec Corporation, Janesville, WI, USA. Dimethyl sulfoxide (DMSO) and triacetin were purchased from Merck KGaA, Darmstadt, Germany. Sodium di-hydrogen orthophosphate-1-hydrate (Minimum Assay 98%), di-sodium hydrogen orthophosphate anhydrous (Minimum Assay Acidimetric 98%), and sodium chloride were acquired from ADWIC, Egypt. Eudragit^®^ Rl 100 was bought from Evonik Operations GmbH, Germany. PURASORB^®^ PDLG 7502 was a kind gift From Corbion Co., Amsterdam, The Netherlands.

### 2.2. Methods

#### 2.2.1. Preparation of Drug-Loaded In Situ-Forming Particulate Formulations (IFPs)

The in situ-forming particulate formulations were prepared using the emulsion method in order to obtain nano-emulsions [24]. The emulsions consisted of two phases: the internal phase, which was prepared by liquifying precise amounts of the particulate-forming agent, and different structural additives in the organic solvent (triacetin), which were incubated in an incubation shaker stirrer (IKA Ks4000ic, Staufen, Germany) at 65 °C ± 0.5 °C (180 stroke/minute) for 12 h (Table 1). Furthermore, a precisely weighed amount of the drug was added to the internal phase, followed by vortexing for one minute. Using a vortex mixer, the accurate and consistent proportions of Captex^®^ GTO and Span^®^ 80 were merged to prepare the external phase. Finally, the emulsion was obtained by simple mixing of the internal and external phases by vertexing for one min.

#### 2.2.2. Design of Experiment (DOE) and Construction of the 2^1^.3^1^ Full Factorial Experimental Design

Design of experiments (DOE) is a systematic and structured approach to planning, conducting, and analyzing experiments or tests in order to obtain the most valuable information from the fewest trials. DOE represents a powerful tool that allows researchers to efficiently optimize processes, understand the influence of various factors, and make data-driven decisions, thus augmenting efficiency, data quality, and robustness and minimizing cost and trial and error while allowing for optimization, which leads to a better data-based decision making [25,26]. DOE was utilized for the generation and evaluation of the obtained models for the formulation of IFPs using Design Expert^®^ 11 software (Stat-Ease, USA). A 2^1^.3^1^ full factorial experimental design was computed to investigate the joint effect of independent formulation variables on the characteristics of the prepared formulations. One factor at two levels and the other at three levels were the two inputs evaluated as independent variables. The two independent factors were (A) the percentage of a particulate-forming agent and (B) the particulate-forming agent’s type. Particle size (PS), Zeta potential, polydispersity index (PDI), mean dissolution time (MDT), percentage drug released after 0.5 h (Q0.5), half-life (T50%), and time required for ninety percent of the drug concentration to be released (T90%) were the computed dependent variables (Table 1).

#### 2.2.3. Characterization of the Prepared IFPs

##### Particle Size and Polydispersity Index (PDI) Determination

Exactly one ml of the formulation was diluted 1:10 with deionized water, followed by one hour of stirring using a magnetic stirrer (Velp-AREC.T F20500051, Velp Scientifica, Usmate Velate, Italy) to produce IFPs. In order to determine the particle size and polydispersity index (PDI) of the formulated IFPs, the prepared sample was centrifuged for 15 min at 15,000 rpm at 4 °C in a refrigerating ultracentrifuge (3-30KS, Sigma Laborzentrifugen, Germany) just before the oily phase was eliminated. Particle size was determined after 1mL of deionized water was used to suspend the separated particles. The mean PS, as well as the vesicle PDI, were determined utilizing ZetaSizer (Nano Zs, Malvern Instruments Limited, Malvern, UK) (n = 3) SD in a dynamic light scattering (DLS) analysis.

##### In Vitro Drug Release Profile and Kinetic Modeling of the Prepared IFPs

In order to investigate the drug’s release pattern from the designed formulation, the donor compartment was a cellulose membrane dialysis bag with dimensions of 7 cm in length, 2.2 cm in width, and a molecular weight cutoff of 12–14,000 Daltons [27]. The dialysis bag was filled with a precisely measured volume (0.5 mL) of drug-loaded IFPs, while exactly 100 mL of phosphate-buffered saline (pH 7.4) were used to mimic the receiving compartment, and the IFPs were incubated at a constant temperature of 37 °C ± 0.5 °C in an incubation shaker (180 rpm). At regular time intervals, 5 mL of the release medium were collected and replaced with fresh medium to maintain the sink conditions. Spectrophotometric analysis was used to determine the amount of encapsulated drug in the withdrawn samples at the previously determined wavelength. The average cumulative drug released percentage was plotted against time, followed by kinetic analysis of the obtained data by computationally fitting the data into various kinetic models, including the Zero, First, and Higuchi diffusion release models, followed by determining the best fit of the release data utilizing linear regression analysis [28,29]. In order to compare the formulations under investigation, various release parameters were calculated. The examined release metrics included mean dissolution time (MDT), percentage of drug released after 0.5 h (Q_0.5_), time required for 25% of the drug concentration to be released (T_25%_), half-life (T_50%_), and time required for 90% of the drug concentration to be released (T_90%_).

#### 2.2.4. Desirability Study

A selected formulation was chosen for additional research using the Design Expert^®^ 11 software’s integrated desirability function. The intended outcomes were to augment MDT, T25%, and T50% and reduce Q0.5 and PS. Only significant models were included.

#### 2.2.5. Investigation of the Effect of Further Variations in Formulation Factors on the Selected Formulation

##### Effect of Adding Some Structural Additives

The selected formulation was subjected to further modification in order to control the initial drug release and enhance the retention time. Eudragit RL, either alone or in combination with Brij 52, was added to the internal phase, and the obtained formulations were characterized to investigate the significance of the addition of these structure additives on the Q0.5 followed by PS if the Q0.5 value was significantly minimized.

##### Effect of Solvent Variation

Further adjustments were made to the chosen formulation from the desirability study section to investigate the effect of different organic solvents on the characteristics of the formulation. DMSO and triacetin were investigated individually and as an equal combination (1:1) as the organic solvent in the internal phase. The obtained formulations were re-evaluated in terms of their Q0.5 followed by PS if the Q0.5 value was significantly minimized.

#### 2.2.6. Characterization of the Optimized Formulation

The optimized formulation was chosen based on better control of the initial release with minor changes in PS for additional examinations.

##### Morphological Study Using Transmission Electron Microscope (TEM)

Using a high-resolution transmission electron microscope (TEM) (HR-TEM)—JEOL2100-USA, Wilmington, DE, USA), optimized IFPs were examined morphologically. To ensure the formation of IFPs, the prepared emulsions were introduced into 10 mL of phosphate buffer of pH 7.4 and then incubated for 24 h in an incubation shaker. The oily phase was separated by centrifugation at 15,000 rpm and 4 °C for fifteen minutes [24]. The morphology of the obtained particulates was examined using TEM with an 80 kV accelerating voltage after they had been dispersed in 1 mL of pH 7.4 phosphate-buffered saline. A drop of the IFPs was positioned on a copper grid that had been coated with carbon, and it was left there for approximately two minutes to adhere. On top of the carbon grid, a drop of phosphotungstic acid solution (2% *w*/*v*) was applied. The prepared sample was air-dried first prior to analyzing the IFP film [30].

##### Rheological Study

A computerized Brookfield rheometer (DV3THB cone/plate rheometer, spindle CPE-40, and RheocalcT software, version 1.1.13 software) (PolyScience model 9006, Niles, IL, USA) was utilized for the viscosity measurement of the chosen IFPs at 25 °C ± 0.2 °C, utilizing a cone and plate setup with a 20 mm diameter/4° angle and a set shear rate (1/s). The rheological characteristics of the prepared formulae were computed by plotting the shear stress versus the shear rate. Farrow’s equation was implemented to investigate the flow pattern:

Log D = N Log S − Log η,

where S stands for the shear stress, and D stands for the shear rate (s^−1^) (Pa). N is the Farrow constant, and η is the viscosity (Pa·s) [31].

##### PBPK Physiological Modeling

Physiologically based pharmacokinetic (PBPK) modeling is a mathematical and computational approach used in pharmacology and toxicology to simulate and predict the behavior of drugs, chemicals, and other substances within the human body. It is a crucial tool in drug development, regulatory approval, risk assessment, and various research areas [18,19].

- Construction of the PBPK Model

PX constructed the PBPK model, which was developed and validated using PK-Sim^®^ version 8.0 (Bayer AG, Leverkusen, Germany). Absorption, distribution, metabolism, and excretion (ADME) process data, physicochemical characteristics, and literature-based clinical pharmacokinetic figures for the drug were acquired from former publications and/or drug databases [32]. The software automatically computed the specific intestinal and organ permeabilities. The renal clearance value was computed to simulate the profile of cumulative excretion as an unchanged form within urine following the ranges presented in Ishizaki et al. [33]. Cellular permeabilities and partition coefficients were computed as Schmitt and PK-Sim^®^ standard methods, respectively [34,35]. The PBPK model was developed for the intramuscular administration protocol of a single 10 mg/kg in the adult population.

- PBPK Model Evaluation

The PBPK model was validated through comparison with the observed clinical data by Calvo et al. after simulating the oral administration of a single dose of 20 mg of PX in adults [36]. The numerical evaluation of the model was carried out by comparing observed to predicted AUC0-24, C_max_, and t_max_ values. The acceptance criterion for the model was set to a two-fold error range. In other words, if the predicted value/observed value (fold error) is in the 0.5–2 range, the PBPK model may be justified [37].

#### 2.2.7. Statistical Analysis of Data

The collected data were displayed as mean ± SD (standard deviation). The computation of the results of the full factorial experimental design was performed using Design-Expert^®^ 11 software (Stat-Ease, Inc., Minneapolis, MN, USA), followed by ANOVA testing to evaluate the statistical significance. The statistical significance level was set at a *p*-value of 0.05 in each experiment.

## 3. Results and Discussion

### 3.1. Preparation of Drug-Loaded In Situ-Forming Particulate Formulations (IFPs)

IFP formulations were prepared using the emulsification method. The resulting nano-emulsions were immiscible liquids that were stabilized with the aid of the appropriate surfactant and/or self-emulsifying oil phase with a typical mean droplet diameter of 500 nm or less. All obtained formulations had a homogenous clear or hazy appearance due to their small droplet size, as opposed to the milky white color of a coarse emulsion [38].

### 3.2. Statistical Analysis of the 2^1^.3^2^ Full Factorial Experimental Design

#### 3.2.1. Effect of Formulation Factors on Average PS and PDI Values of the Prepared IFPs

**Figure 1:**
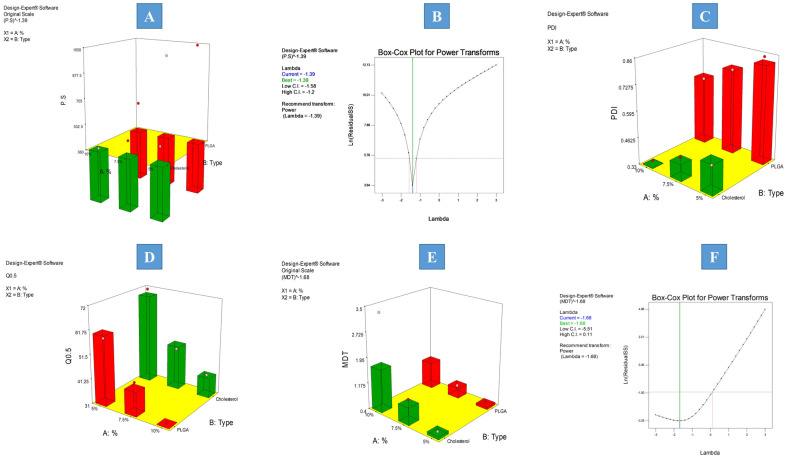
Example of 3D-response surface plots for the effect of formulation factors on (**A**) PS, (**B**) Box–Cox transformation for PS, (**C**) PDI, (**D**) Q0.5, (**E**) MDT, and (**F**) Box–Cox transformation for MDT.

To determine the level of significance of the examined independent factors on the particle size and PDI of the formulations, an ANOVA test was conducted. The measured response values are presented in Table 1, and the model regression analysis is presented in Table 2. The particle size ranged from 363.5 ± 21.67 to 1043 ± 98.26 nm, and the PDI ranged from 0.347 ± 0.017 to 0.854 ± 0.064. The two models showed good correlation between the values of the R^2^ (0.9999 and 0.9891, for PS and PDI, respectively), adjusted R^2^ (0.9997 and 0.9728, for PS and PDI, respectively), and predicted R^2^ (0.9990 and 0.9022, for PS and PDI, respectively), as well as the adequate precision of values (187.885 and 18.163, for PS and PDI, respectively), which guarantees the adequacy of the constructed model and ensures that the model may be used to investigate the entire design space. The PDI model results were sufficiently satisfactory with no need for further transformation, while the PS model required further transformation, as evident from the Box–Cox diagnostic, as presented in Figure 1B. Results revealed that both the percentage of particulate-forming agent (A) and the type of particulate-forming agent (B) significantly (*p* = 0.0004 and <0.0001, respectively) influenced the obtained values of particle size. The increase in the percentage of the particulate-forming agent significantly decreased the particle size, as presented in Figure 1A. This can be simply due to the better availability of the particulate building block with the possibility of better crosslinking leading to the formation of denser core smaller particulates. On the other hand, the cholesterol (CHL)-based particulates exhibited a significantly smaller particle size compared to the PDLG particulates. This may be attributed to the CHL imparting rigidity to the obtained particulates and decreasing the fluidization of the particles with a minimization of the surface free energy, all of which results in smaller-sized particulates. Moreover, the increased lipophilicity accompanied by the change in the type of particulate forming agent from PDLG to CHL, which in turn slowed down the diffusion of the internal phase solvent and consequently the deposition of the particles may have resulted in the formation of denser, uniform, and smaller particles. This is consistent with the outcomes that were described by Saberi et al. [39].

Regarding the PDI, only the type of particulate-forming agent significantly (*p* = 0.0061) influenced the PDI of the resulting particles, as presented in Figure 1C. Changing the particulate-forming agent from PDLG to CHL significantly decreased the PDI. This is in good agreement with the PS results, where the observed decrease in PS was accompanied by better homogeneity in the obtained size distribution, resulting in monodisperse systems with lower PDI values. These results are comparable to the results observed by Kumar et al. [40].

#### 3.2.2. Effect of Formulation Factors on In Vitro Release Parameters

In vitro release study is one of the most fundamental studies for most controlled release systems. It is a great way to eliminate systems with release profiles that are undesirable. The effectiveness of in vitro tests for evaluating the finished systems’ quality is extremely important.

**Figure 2:**
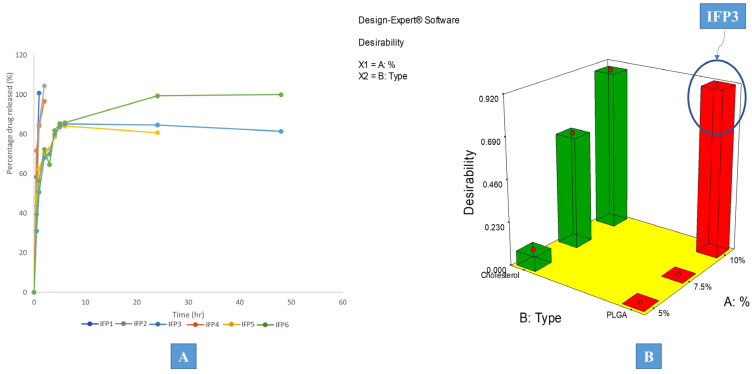
(**A**) Release profile of the prepared IFPs and (**B**) the desirability study marked with the chosen formulation.

As shown in Figure 2A, all formulations displayed a two-phased release pattern, exhibiting an initial rapid release phase, then a more extended-release stage follows. As soon as the formulation meets the dissolution medium, diffusion of the internal phase solvent takes place through the external oily phase, causing the particulates to deposit and solidify, trapping the drug into its core. The existence of some drug that was not trapped into the core of the produced particulates and is free to be released more rapidly than the drug that is entrapped, is what causes biphasic release [41]. In order to evaluate the distinctions between the prepared formulations, various release parameters were computed. The studied release parameters were mean dissolution time (MDT), percentage of drug released after 0.5 h (Q_0.5_), time required for 25% of the drug concentration to be released (T_25%_), half-life (T_50%_), and time required for 90% of the drug concentration to be released (T_90%_). Only the constructed models for mean dissolution time (MDT) and percentage drug released after 0.5 h (Q_0.5_) proved significant. Insignificant models were excluded from the study.

The average cumulative drug release percentage was plotted versus time, and the release data were kinetically analyzed by substituting the obtained release data into various kinetic models, including the First, Zero, and Higuchi diffusion release models, utilizing linear regression analysis to find the release data’s best fit. Followed by confirmation of the obtained results using the Korsmeyer–Peppas equation. All formulations exhibited first-order release kinetics, which is common in many particulate systems [42].

##### Effect of Formulation Factors on Q_0.5_

To determine the level of significance of the examined independent variables on Q_0.5_, an ANOVA test was conducted. The values of the measured responses are shown in Table 1, while the model regression analysis is presented in Table 2. The constructed model showed a good correlation between the values of the R^2^ (0.9797), adjusted R^2^ (0.9492), and predicted R^2^ (0.8171), as well as the adequate precision of value 14.411, which assures the adequacy of the model. Results showed that the Q_0.5_ values ranged from (31.10 ± 2.80 to 71.65 ± 5.42) and only the percentage of particulate forming agent (A) significantly (*p* = 0.0227) influenced the obtained values of (Q_0.5_). The change in the percentage of the particular forming agent from the lower level to the higher level significantly decreased the Q_0.5_ values, which reflects the drug’s delayed release and the management of the formulation’s well-known burst effect, as shown in Figure 1D. Additionally, since PDLG and cholesterol are the key particle producers, an increase in their percentage results in the formation of more particles; thus, more drug was entrapped within the formed particles. This decrease in the amount of free drug resulted in a significant decrease in Q_0.5_, representing better control of the drug release pattern.

##### Effect of Formulation Factors on the Mean Dissolution Time (MDT)

Utilizing the ANOVA test, the level of significance of the independent factors on the MDT values was computed as shown in Table 1 and Table 2 and ranged from 0.46 ± 0.03 to 3.32 ± 0.28 h. Applying the Box–Cox diagnostic test, power transformation was implemented to augment the sensitivity of the constructed model, as presented in Figure 1F. Results showed that the percentage of particulate forming agent (A) significantly (*p* = 0.0138) augmented the MDT values, as shown in Figure 1E. The previously observed significant control of Q_0.5_ was attributed to the increase in PLGA and cholesterol concentrations, which accelerated the standard rate of IFPs deposition. Subsequently, the significantly increased portion of the entrapped drug reduced the initial release, revealed in the significant minimization in Q_0.5_. All of which adds up to the total delay in medication release and the notable rise in MDT. This facilitated the total delay in the release of the drug and the notable rise in MDT value.

### 3.3. Desirability Study

IFP3 was recognized as the chosen formulation for additional investigation based on the desirability study implemented using Design^®^ Expert desirability function with the target criteria of minimizing PS, PDI, and Q_0.5_ and maximizing MDT, as shown in Figure 2B. Only significant models were included.

### 3.4. Further Investigation of the Effect of Formulation Factors on the Selected Formulation

#### 3.4.1. Effect of Some Structural Additives

**Figure 3:**
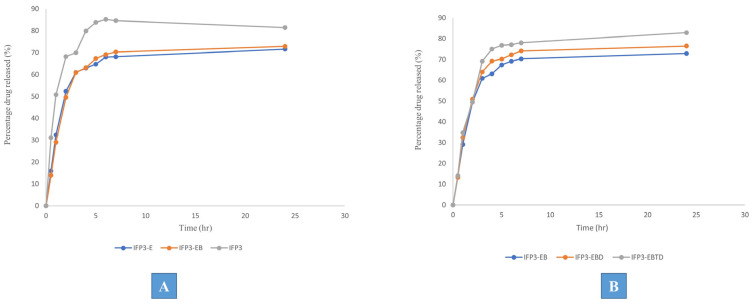
Release profiles of the chosen formulation IFP3 against modified formulations with (**A**) structural additives and (**B**) different solvents.

Further modifications were implemented into the optimized formulation IFP3 in terms of minimizing the initial release of the drug and maximizing the retention time. Eudragit RL was added as a structural additive in the internal phase with a concentration of 2.5% either alone (IFP3-E) or in combination with 2.5% Brij 52 (IFP3-EB). The release profile showed that the combination between Eudragit RL and Brij 52 significantly decreased the initial dug release represented in minimization of Q_0.5_, which was 15.95 ± 1.32 and 13.95 ± 0.9% for IFP3-E and IFP3-EB, respectively, which are both significantly lower than IFP3, which had a Q_0.5_ value of 31.10 ± 2.80%, as shown in Figure 3A. This may be due to the expected physical interaction of the O–H group of Brij 52 and the C=O group of the drug that may suggest the development of a new hydrogen bond between the PX and Brij 52, which may have augmented the drug encapsulation within the deposition phase of the IFPs. Moreover, the presence of Eudragit RL may have increased the viscosity of the injected solution favorably, slowing down the diffusion of the internal phase into the surrounding medium and the deposition of the particulates allowing the encapsulation of more drug, which has significantly controlled Q_0.5_. This is in correlation with the findings of Yacoub et al. [43]. The PS of IFP3-EB was 573.92 ± 23.5 nm, which was insignificantly (*p* > 0.05) different from IFP3.

#### 3.4.2. Effect of Solvent Variation

For many gases, synthetic fibers, paint, hydrocarbons, salts, and natural products, DMSO is a useful industrial and laboratory solvent. It is stable at high temperatures, aprotic, and relatively inert. Furthermore, DMSO has low acute and chronic toxicity. High concentrations of test organisms exposed via contact, ingestion, or inhalation repeatedly show low toxicity. It was noticed that the use of DMSO enhanced the solubility of the particulate-forming agents either alone (IFP3-EBD) or in combination with triacetin in the ratio 1:1 (IFP3-EBTD). The results showed the insignificance (*p* > 0.05) of changing the solvent from triacetin to DMSO in terms of its effect on the release profile of the prepared formulations, as shown in Figure 3B. Therefore, IFP3-EBD was chosen for further investigations based on the proven safety margin of DMSO compared to triacetin [44]. The PS of IFP3-ED was 579.12 ± 13.55, which was insignificantly (*p* > 0.05) different from IFP3-EB in spite of the difference in polarity between DMSO and triacetin. This may be attributed to the presence of Brij 52 with its large hydrophilic head that may have hindered the expected rapid diffusion of DMSO into the surrounding media and conserved the PS.

### 3.5. Characterization of the Optimized Formulation

#### 3.5.1. Transmission Electron Microscopy (TEM)

**Figure 4:**
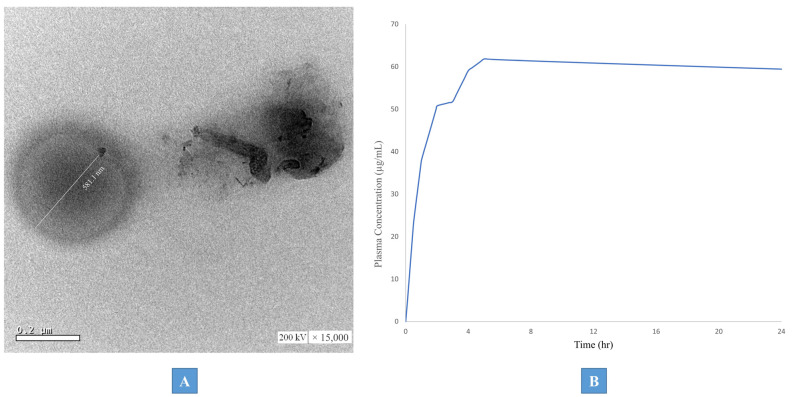
(**A**) Transmission electron micrograph (TEM) of the optimized formulations (IFP3-EBD). (**B**) PBPK simulated PX plasma concentration–time curves following IM application of IFP3-EBD.

The morphological pattern of the optimum formulation IFP3-EBD was identified using TEM. As shown in Figure 4A, the generated photographs demonstrated the deposition of uniformly spherical particulates with a dense core. This may be explained by the particulate formation mechanism, which depends on the solvent diffusing into the aqueous surroundings and the particulates depositing there. The assembly of dense particulates may have occurred as a result of the Brij 52 characteristic large polar head, delaying the diffusion of DMSO into the release medium.

#### 3.5.2. Rheological Study

Rheology of the injectable formulation represents a crucial characteristic as it may hinder the ease of administration as well as cause pain upon application. IFP3-EBD exhibited a pseudoplastic flow, proved by the computed n value from Farrow’s equation (n = 3.95). This flow ensures ease of application with minimal pain due to its decreased viscosity upon applying shear, which is favored in these types of formulation.

#### 3.5.3. PBPK Physiological Modeling

Prior to the adult PX pharmacokinetic parameters’ prediction following the intramuscular (IM) application of the optimum IFP3-EBD formulation, the constructed PBPK model was validated against the reported clinical data by Calvo et al. [36]. The model was verified by contrasting the T_max_, C_max_, and AUC_0-24_ of PX after a single oral dosage of 20 mg with the corresponding published pharmacokinetic values by Calvo et al. As shown in Table 3, the findings showed that the mean predicted/observed ratios for T_max_, C_max_, and AUC_0–24_ were 1.3, 0.9, and 0.6, respectively. This could verify the accuracy of the model that was selected and the modeling software that was being evaluated.

The PBPK model-simulated plasma concentration–time curve of PX following the IM application of the optimized formulation in adults is presented in Figure 4B, while the predicted pharmacokinetic parameters are presented in Table 4. Results revealed that the C_max_ value of PX following the IM application of IFP3-EBD (10 μg/mL) was significantly higher than the corresponding mean value (2.28 μg/mL) observed with the reference oral administration (20 mg). At the same time, the AUC_0–24_ of the IFP3-EBD was 1383.03 compared to a value of 78.7 (μg·h/mL) for the observed oral formulation with a relative bioavailability value of 1757%. Finally, the T_max_ was 5 and 4 h for IFP3-EBD and the oral formulation, respectively. This obvious augmentation in the pharmacokinetic profile of IFP3-EBD compared to the oral formulation is attributed to the remarkable components of the IM formulation as well as the nan-range of the obtained particulates upon injection. Brij 52, as a surfactant, has the ability to enhance the biological absorption of the encapsulated drugs via the liquefaction of the biological membranes and the loosening of their tight junctions, allowing for better penetration of the drug into the bloodstream. Moreover, Brij 52 imparts more flexibility to the basically soft lipidic nano-particulates, thus facilitating their penetration. Additionally, these particulate systems serve as nano-reservoir systems that release the drug in a continuous and controlled manner. Finally, the negatively charged particulates are thought to have a lower clearance rate than that of the neutral ones. This is highly reflected by the maximized C_max_ and AUC_0–24_ values of IFP3-EBD. This is in line with the reported observations of Sharma et al. [45]. The comparably higher T_max_ of the IM formulation may be attributed to the sustained release pattern of the drug from the IFP3-EBD nano-particulates.

## 4. Conclusions

Design of experiments was adopted for the design, characterization, and optimization of in situ-forming particulates (IFPs) for the IM administration of piroxicam via designing a full factorial experimental approach where the effect of the different formulation variables on the characteristics of the obtained IFPs was studied. The selected formulation was further investigated for the influence of the addition of some structural additives to augment the kinetic profile of the drug release. The optimized formulation (IFP3-EBD) presented favorable rheological features with the formation of spherical dense particulates upon injection showing minimal aggregates. The study of the release profile proved the extended-release behavior of IFP3-EBD endorsed with the virtual investigation of its biological efficacy using PBPK physiological modeling. The adopted physiological model was proved reliable upon its correlation to the literature-based clinical data. The optimized formulation exhibited an augmented biological profile with a relative bioavailability of 1757% compared to the literature data. These results demonstrated the ultimate importance of the close control of the different formulation factors for the significant effect of their variation on the characteristics of the obtained drug delivery systems. The invented IFPs proved their potential for efficient piroxicam IM delivery as well as the reliability of the PBPK physiological modeling in the prediction of the biological performance of novel formulations in a cost-effective, comprehensive manner.

## Figures and Tables

**Table 1:** Dependent and independent variables of the 2^1^.3^1^ full factorial experimental design of the piroxicam loaded in situ-forming particulate formulations.

Formulae	Independent Factors		Dependent Factors *								
	Percentage of Particulate Forming Agent	Type of Particulate Forming Agent	Particle Size Mean ± SD (nm)	PDI Mean ± SD (nm)	Zeta Potential Mean ± SD (mV)	Q_0.5_ ± SD (h)	K ± SD (h^−1^)	T_25%_ ± SD (h)	T_50%_ ± SD (h)	T_90%_ ± SD (h)	MDT ± SD (h)
IFP1	5%	PLGA	1043 ± 98.26	0.854 ± 0.064	−11.6 ± 0.81	58.4 ± 3.31	2.20 ± 0.14	0.13 ± 0.016	0.32 ± 0.017	1.51 ±0.12	0.46 ± 0.03
IFP2	7.5%	PLGA	951 ± 87.10	0.751 ± 0.041	−11.4 ± 0.92	43.65 ± 2.34	1.53 ± 0.12	0.19 ± 0.013	0.45 ± 0.020	3.60 ± 0.42	0.68 ± 0.04
IFP3	10%	PLGA	569 ± 34.21	0.663 ± 0.036	−18.4 ± 1.56	31.10 ± 2.80	0.50 ± 0.06	0.58 ± 0.041	1.40 ± 0.160	4.66 ± 0.51	1.24 ± 0.14
IFP4	5%	Cholesterol	470.5 ± 23.89	0.452 ± 0.035	−12.6 ± 0.76	71.65 ± 5.42	2.27 ± 0.19	0.13 ± 0.018	0.31 ± 0.024	4.17 ± 0.36	0.48 ± 0.06
IFP5	7.5%	Cholesterol	462 ± 39.74	0.426 ± 0.024	−12.5 ± 1.10	47.70 ± 3.90	0.64 ± 0.08	0.45 ± 0.032	1.10 ± 0.250	1.05 ± 0.16	1.04 ± 0.09
IFP6	10%	Cholesterol	363.5 ± 21.67	0.347 ± 0.017	−12.8 ± 1.07	39.45 ± 1.23	0.55 ± 0.02	0.52 ± 0.047	1.26 ± 0.19	1.02 ± 0.22	1.32 ± 0.28

* n = 3. PDI: polydispersity index; K: release rate constant; T90%, T50%, and T25%: time required for 90, 50, and 25% of the drug to be released, respectively; MDT: mean dissolution time; Q0.5: percentage drug released after 0.5 h. Internal phase contains 2 mg of piroxicam in all formulations stabilized by 0.1% tween 80.

**Table 2:** Model parameters of the 2^1^.3^1^ full factorial experimental design of the piroxicam-loaded in situ-forming particulate formulations (IFPs).

Model Parameters	PDI Mean (nm)	Particle Size Mean (nm)	Q0.5 (h)	MDT (h)
Model Type	Main Effects	Main Effects	Main Effects	Main Effects
R^2^	0.9891	0.9999	0.9797	0.9868
Adjusted R^2^	0.9728	0.9997	0.9492	0.9670
Predicted R^2^	0.9022	0.9990	0.8171	0.8811
Adequate Precision	18.163	187.885	14.411	16.929
Final Equation in Terms of Coded Factors	PDI = +0.58 +0.071 * A [1] +6.333 × 10^−3^ * A [2] −0.17 * B	(P.S)^^−1.39^ = +1.585 × 10^−4^ −3.019 × 10^−5^ * A [1] −2.335 × 10^−5^ * A [2] +6.371 × 10^−5^ * B	Q0.5 = +48.66 +16.37 * A [1] −2.98 * A [2] +4.28 * B	(MDT)^^−1.68^ = +1.80 +1.77 * A [1] −0.38 * A [2] −0.29 * B

PDI: polydispersity index; Q0.5: percentage drug released after 0.5 h; MDT: mean dissolution time; R2: squared regression coefficient.

**Table 3:** Results for the development and validation of the piroxicam PBPK model.

Reference	AUC_0-24_ (μg·h/mL)			C_max_ (μg/mL)			T_max_ (h)		
	Observed	Predicted	Fold Error *	Observed	Predicted	Fold Error *	Observed	Predicted	Fold Error *
Calvo et al. [36]	78.7	45.4	0.6	2.28	2	0.9	4	5.25	1.3

* Fold error indicates predicted value divided by observed value (predicted/observed). AUC_0−24_: area under the plasma concentration–time curve; C_max_: maximum plasma concentration; t_max_: time required to reach maximum plasma concentration.

**Table 4:** Physiologically based pharmacokinetic model simulating piroxicam pharmacokinetic parameters following intramuscular administration of IFP3-EBD at 10 mg/kg dose in adults.

AUC_0-24_ (μg·h/mL)	C_max_ (μg/mL)	T_max_ (h)
1383.03	61.79	5

AUC: area under the plasma concentration–time curve; C_max_: maximum plasma concentration; T_max_: time required to reach maximum plasma concentration; IFP3-EBD: in situ-forming particulate formulation containing Eudragit RL and Brij 52 as structural additives and DMSO as solvent.

## Data Availability

The datasets generated during and/or analyzed during the current study are available from the corresponding author upon reasonable request.
